# Current Status of Amino Acid-Based Permeation Enhancers in Transdermal Drug Delivery

**DOI:** 10.3390/membranes11050343

**Published:** 2021-05-07

**Authors:** Rui Pereira, Sandra G. Silva, Marina Pinheiro, Salette Reis, M. Luísa do Vale

**Affiliations:** 1 LAQV-REQUIMTE, Departamento de Química e Bioquímica, Faculdade de Ciências, Universidade do Porto, Rua do Campo Alegre s/n, 4169-007 Porto, Portugal; up201502919@fc.up.pt (R.P.); sandra.silva@fc.up.pt (S.G.S.); 2 LAQV-REQUIMTE, Departamento de Ciências Químicas, Faculdade de Farmácia, Rua de Jorge Viterbo Ferreira, 228, 4050-313 Porto, Portugal; mpinheiro@ff.up.pt (M.P.); shreis@ff.up.pt (S.R.)

**Keywords:** amino acids, amphiphiles, chemical permeation enhancer, transdermal delivery, skin

## Abstract

Transdermal drug delivery (TDD) presents many advantages compared to other conventional routes of drug administration, yet its full potential has not been achieved. The administration of drugs through the skin is hampered by the natural barrier properties of the skin, which results in poor permeation of most drugs. Several methods have been developed to overcome this limitation. One of the approaches to increase drug permeation and thus to enable TDD for a wider range of drugs consists in the use of chemical permeation enhancers (CPEs), compounds that interact with skin to ultimately increase drug flux. Amino acid derivatives show great potential as permeation enhancers, as they exhibit high biodegradability and low toxicity. Here we present an overview of amino acid derivatives investigated so far as CPEs for the delivery of hydrophilic and lipophilic drugs across the skin, focusing on the structural features which promote their enhancement capacity.

## 1. Introduction

The act of administering a drug is one of the most important steps in medical practices, and the administration method greatly influences the drugs’ success. Transdermal drug delivery (TDD) offers numerous advantages over other conventional routes of administration, such as oral and parenteral administration. In fact, the direct delivery of the drugs to the blood circulation avoids gastrointestinal and liver first-pass effects, allowing the use of drugs that are not suitable for oral medicines due to metabolism or common side effects [1,2]. Furthermore, compared to parenteral administration routes, TDD is painless, does not generate as much hazardous medical waste, and presents less risk of disease transmission by needle re-use, a special concern in developing countries. TDD is non-invasive and easy-to-use and generally presents enhanced patient compliance (no needle phobia, less frequent administration). At last, it is the only administration method that provides a sustained effect during long periods [1,3]. Thus, TDD can overcome the low bioavailability of many oral drugs, the pain and inconvenience of injections, and the limited controlled release of both methods. Despite the advantages, TDD has not yet achieved its full potential, and its application is limited only to a small number of drugs with low molecular masses and a favorable lipophilic behavior, whose skin permeability coefficients are sufficiently high to achieve clinically active plasma levels [4]. The biggest challenge in TDD systems is to overcome the skin barrier effect. The skin constitutes an excellent protective physiological barrier since it prevents the entrance of xenobiotics into the body and minimizes water loss having, therefore, low permeability for the penetration of foreign molecules [3]. The Stratum corneum (SC), the outermost layer of the skin, constitutes the primary barrier to drug penetration. The low permeability of the SC is due not only to its unique lipid composition but also to the unique structural organization of the lipids [5,6].

Several approaches have been made to develop novel TDD systems with increased drug permeation across the SC [7,8,9,10]. One of the most studied strategies to circumvent the SC and thus improve transdermal permeation consists in the use of chemical permeation enhancers (CPEs), compounds able to interact with the SC layer and induce a temporary, reversible increase in skin permeability. To date, a wide variety of compounds, pertaining to different classes, which can be further combined into six different groups, have been identified as CPEs: alcohols and polyols, lactams and their analogs, steroids, terpenes, and fatty acids, surfactants, esters, and ethers and miscellaneous others [11]. In this review, the current status quo of amino acid-based chemical enhancers will be addressed. Amino acid-based CPEs constitute a promising class of biocompatible compounds with low cytotoxicity, high activity, and reversibility. The main objective is to list and discuss the characteristics of amino acid derivatives as enhancers in TDD.

## 2. Skin Structure and Skin Permeation Routes

**Figure 1:**
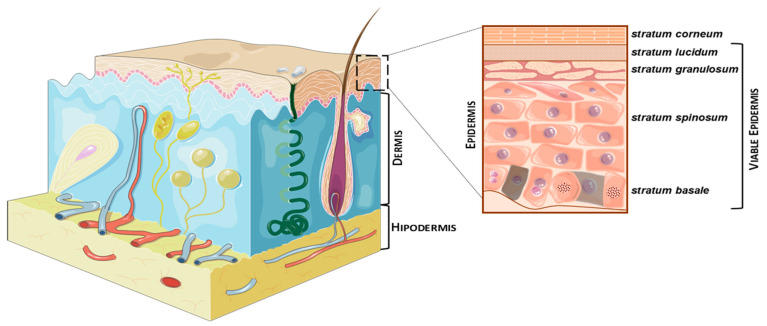
The structure of the skin; images from Servier Medical Art.

The skin is the largest human organ, responsible for about 15% of the total body weight, covering a surface area of 2 m^2^ in adults [1,12]. It is a multilayered structure composed of three larger layers: epidermis, dermis, and hypodermis (Figure 1). The epidermis, the uppermost layer of the skin, is organized into five Strata: the stratum corneum (SC), the outermost layer and also known as the non-viable epidermis which is 10–20 μm thick, and the stratum lucidum (SL), stratum granulosum (SG), stratum spinosum (SS), and stratum basale (SB), which together represent the viable epidermis, measure 50 to 100 μm and are avascular [12,13,14]. Deeper into the skin is the dermis, with a thickness of about 1–2 mm. The dermis is composed of fibroblasts that compose the extracellular matrix, alongside high concentrations of collagen, elastin, and glycosaminoglycans. It is rich in vascular circulation, necessary for drug absorption.

The SC constitutes the main barrier to drug permeation. It is mostly composed of corneocytes, separated by an intercellular lipid domain, generally known as the “brick and mortar” model [15,16,17,18,19]. The composition of the intercellular lipid mixture differs significantly from other cellular membranes: it is particularly rich in ceramides (40–50% wt%), fatty acids (7–13%), and cholesterol (20–33%), with minor quantities of cholesterol sulfate (2–5% wt%), and is organized in bilayer arrays, thus forming an impermeable barrier to drug diffusion [16,20,21,22].

**Figure 2:**
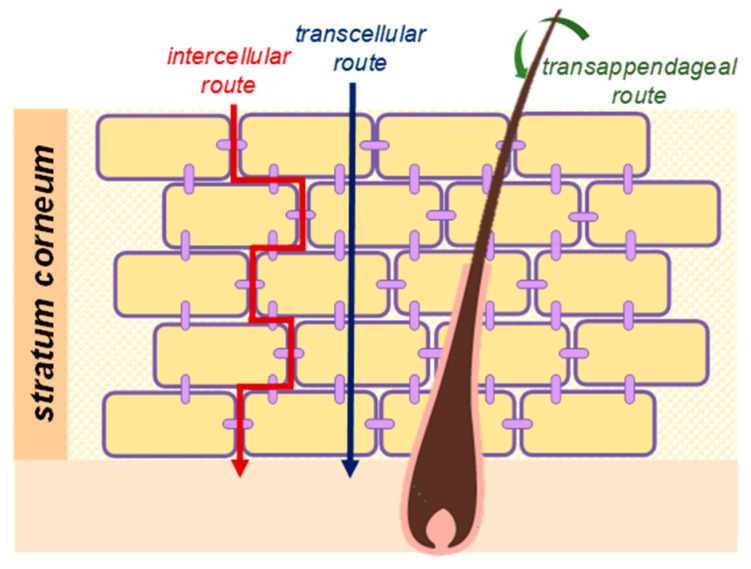
Drug permeation pathways through the skin.

A drug or molecule applied to the skin surface has three potential pathways to penetrate across the SC: the transappendageal route (via hair follicles and associated sebaceous glands), the transcellular route (diffusion across corneocytes), and the intercellular route (diffusion across the continuous epidermis) (Figure 2) [14,17,23,24].

The transappendageal approach encompasses penetration through hair follicles or sweat ducts. The contribution to percutaneous transport is often considered secondary since appendages account for only about 0.1% of the total surface of the skin [25]. Nonetheless, this route can be a significant positive contributor to drug permeation, especially for highly hydrophilic molecules and ions as well as large polar molecules and polymers with low diffusion coefficients, which are not able to freely penetrate across the SC [26,27]. The transcellular route is identified as the polar path through the SC. It involves a complex process of drug partitioning between the hydrophilic environment of the corneocytes and the lipophilic domains of the intercellular lipid matrix, which turns this route very resistant to drug permeation, albeit it may still be the choice for polar drugs [28]. The intercellular route provides a continuous pathway through the intercellular lipid domain around corneocytes [17]. In this case, the polar head groups of the lipids favor the permeation of hydrophilic molecules, while for lipophilic drugs, transport can occur through the lipid tails [14,28]. Although the intercellular route is recognized as the main pathway for skin penetration, in part as a consequence of the lipophilicity of most of the drugs, it is also globally accepted that, in most cases, a combination of these pathways is responsible for the observed permeation across the skin.

The processes involved in transdermal permeation, such as the release of the permeant from the dosage form, its diffusion into and through the SC, and its partitioning to the epidermal environment, depending on the physicochemical characteristics of the permeant as well as on the specific characteristics of the skin on the application site. The rate of drug permeation can be described in a simplistic way using the steady-state flux (*J_ss_*) or Fick’s law of diffusion, which represents the cumulative mass of drug passing per unit area through the skin, *m*, per unit time (Equation (1)) [3]:

(1) $$J_{ss}=\frac{dm}{dt}=\frac{DC_{v}K}{h}\;$$

where *C_v_* is the concentration of drug in the formulation, *K* is the partition coefficient of the drug between the SC and the formulation, *D* is the diffusion coefficient of the drug in the skin, and *h* is the diffusional path length.

Based on Equation (1), for a molecule to present high permeation potential, it should possess:

♦ Low molecular mass (<500 Da);
♦ Adequate solubility in oil and water (*C_v_* is large);
♦ Moderate lipophilicity (*K* = 1–5, a too-large value may inhibit clearance by viable tissues);
♦ Low melting point (<250 °C); melting points reflect the non-covalent interactions between drug molecules and relate to drug solubility in the SC.

Whenever the physicochemical properties of a molecule do not allow its permeation in therapeutically relevant doses, enhancement strategies must be considered.

## 3. Permeation Enhancement Strategies and Mechanisms

The main challenge of TDD is to design a system that can successfully deliver any drug through the skin. Over the past decades, extensive research has been carried out in this area, and several methods for enhancing the drug delivery into/through the skin have been developed. These methods can be mainly divided into chemical and physical approaches [29,30].

Chemical methods are designed to increase the natural driving force of molecules through the skin, their diffusion coefficient, and their solubility in the SC by applying chemicals. This can be accomplished by improving the permeation capacity of the formulation, using drug vehicles (e.g., vesicular systems, microemulsions, and nanoparticles), by improving the permeation capacity of the drug, which can be achieved by increasing the drug’s concentration to favor its partitioning—there are several strategies to solubilize high amounts of drugs using, e.g., ion pairs, eutectic mixtures, supersaturated systems, among others [31]—or by modifying the SC, as to reduce the natural skin resistance to penetration, using CPEs [3,30].

**Figure 3:**
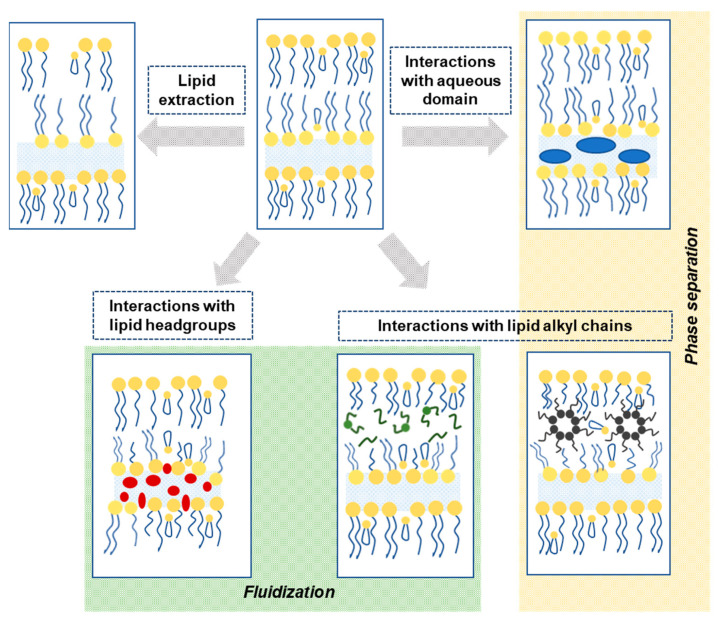
Schematic representation of possible ways by which permeation enhancers may interact with the SC intercellular lipid domain.

The mechanisms by which CPEs affect skin permeability are not yet totally understood, but the development of novel analytical methods in the last decades is giving a great push towards this challenge. However, the Lipid-Protein-Partitioning (LPP) theory presented by Barry B.W. [28,32] assumes that CPEs can act by one or more of three mechanisms: (1) disruption of the intercellular lipid domains, (2) interaction with the intracellular protein domains, or (3) increasing the partitioning of a drug into the skin. In the intercellular lipid phase, enhancers may break down lipid packing and/or fluidize the lipid chains, creating a more permeable domain (Figure 3). This can be achieved by interacting at three different regions associated with the lipid bilayers: Hydrophilic CPEs can interact with the polar head groups, altering the hydration spheres and thus disturbing lipid packing. This interaction results in a more fluid lipid domain and thus enhanced flux of hydrophilic/lipophilic penetrants. Solvents, e.g., ethanol and dimethylsulfoxide, may act by extracting lipids from the bilayer, creating holes in the lipid phase. Interaction at the aqueous domain of the lipid bilayer may provoke swelling, increasing the domain volume and changing the bilayer thickness. Lipophilic CPEs, on the other hand, are able to insert between the hydrophobic chains, disturbing the lipid packing and thus increasing lipid fluidity [33].

In the intracellular protein domain, CPEs interact with keratin, changing its conformation and opening water channels in the cells, facilitating the transport of hydrophilic molecules. They may also interact with corneodesmosomes, altering the interconnections between corneocytes. At last, CPEs can be combined with a co-enhancer, water, or a mixture of both to change the SC solution properties, increasing partitioning of the drug into the skin [32].

However, the LPP theory does not explain why a given enhancer cannot increase permeability for all drugs, why the activity of an enhancer is concentration-dependent, or why some perform best at lower rather than at higher concentrations. More recently, Haq, A. and co-workers proposed a new theory to define the action of penetration enhancers: The Solubility-Physicochemical-Thermodynamic (SPT) theory [34]. According to this theory, the drug flux depends primarily on the thermodynamic activity of the enhancer and not on its concentration. Moreover, it assumes that drug flux is mostly affected by two parameters: the solubility of the drug/enhancer in the formulation and in the SC environment and the physicochemical interactions between the drug and enhancer. For both these parameters, there are optimum values, and the mechanism of action of an enhancer is always drug-specific.

Physical methods are based on the use of external forces to promote the permeation of drugs into the SC or to bypass the SC directly. They are divided into electrical-assisted methods (iontophoresis, electroporation, and radiofrequency), mechanical methods (microneedles, abrasion, perforation, and skin stretching), and other physical methods (ultrasounds, laser radiation, and thermophoresis). When comparing chemical to physical methods, the latter surpass efficacy and safety [7,8,35,36]; however, chemical enhancers present several advantages for the industry and consumer. TDD systems based on CPEs are generally cheaper than physical equipment, and their properties can be adjusted in detail through chemical modifications. Furthermore, chemical enhancers can be formulated into creams, gels, and patches, which, from a consumer point of view, are attractive strategies as they are easy to use, non-invasive, and self-administrable [30,37]. More recently, physical and chemical approaches have been merged to create a hybrid approach in the same formulation to enable new high-impact applications [38].

## 4. Chemical Penetration Enhancers (CPEs)

Chemical penetration enhancers (CPEs) are compounds able to interact with the SC layer and induce a temporary, reversible increase in skin permeability. For a compound to have potential as a CPE, it must fulfill some of the following requirements [30]:

♦ It should be non-toxic, non-irritating, and non-allergenic;
♦ It should be specific to drug permeation without any other bioactivity;
♦ It should present a rapid onset of effect, foreseeable and reproducible;
♦ Its effect should be reversible when removed from the application site;
♦ It should present unidirectional activity, enabling drug permeation without water loss from inner tissues;
♦ It should be biocompatible with drug and excipients;
♦ It should have cosmetic acceptance from consumers.

In the last decades, many compounds with permeation enhancement capacities have been developed, yet only first-generation CPEs are on the market (e.g., ethanol, oleic acid, or propylene glycol). This lack of new molecules in marketed products may be attributed to several factors: the new molecules do not improve the drug flux significantly more than the current ones used; the new molecules significantly improve drug permeation but present safety problems [8,35]; the effects of new molecules are difficult to predict, and their mechanisms of action are not fully understood.

### 4.1. Classification of CPE

The vast diversity of currently available enhancer molecules calls for a systematic classification to allow some organization within this group of compounds (Table 1). However, there are several parameters that can be considered when attempting to categorize them. Classifications based on their (a) origin, natural [39,40,41,42,43], synthetic [44,45,46,47,48], or semi-synthetic [44,49], and on their (b) chemical structure, alcohols [50,51,52,53], fatty acids [51,54], surfactants [48,55], esters [46,51,56,57,58,59,60,61,62], amides [45,62], and sulfoxides [63,64] are the most common.

### 4.2. Structure-Activity Relationships

As can be seen from Table 1, many of the permeation enhancers are amphiphilic molecules with a polar head group and a lipophilic chain. According to the LPP theory, this structure should lead to strong interactions with the lipid bilayers of the SC since these compounds disrupt the lipid organization, reducing chain order and increasing fluidity [65,66]. In fact, the length of the alkyl chain has significant importance for the effect on the lipid barrier, with 10–12 carbon alkyl chains presenting the best profile. These chains are shorter than those of the fatty acids found in the lipid matrix of the SC (18 carbons or more), free or attached to ceramides. So, the shorter amphiphiles will incorporate into the lipid packing, forming pores under the shorter chains, thereby reducing the interaction between ceramides and increasing fluidity [48,67,68]. Unsaturation of the alkyl chain also has a beneficial effect on the enhancing capacity, as the kink induced in the structure of the alkyl chain by the presence of a *cis*-double bond will also lead to fluidization of the lipid domain. Here, alkyl chain lengths of 18–20 carbon atoms lead to the best performances, with oleic acid as the most studied example [69,70].

The effect of the polar head structure is less pronounced than that of the hydrophobic counterpart. However, since the head group is expected to be located in the hydrophilic region of the lipid bilayers, it should be small enough to be incorporated in the bilayer but different from the ceramide head group to cause packing irregularities [30,68]. Moreover, polar groups have the ability to establish hydrogen bonds, which allow increasing the hydration sphere, leading to a higher aqueous phase between each lipid bilayer, which improves drug transport for hydrophilic molecules. The increase in water in the SC can also lead to an additional permeation enhancement. Water is a recognized powerful natural CPE, and even though the mechanism of action is not fully understood, it helps permeation of both lipophilic and hydrophilic molecules [65]. Hydrogen bonds are also directly responsible for lipid extraction from the lipid domain, another way of causing holes in the packing.

Another structural property that may have a significant role in the potency of enhancers is the chirality of the head group. The importance of stereochemistry in drug R&D is well known. In fact, most biological membranes have a chiral environment that can influence many important aspects of drug penetration, such as melting point, solubility, and stability, which may vary with stereochemical purity. Many topical formulations also use excipients with known chirality, such as cellulose. Despite the evidence that drug chirality may have consequences on drug permeation, there is not much research on the influence of chirality of enhancers and their interaction with chiral drugs, enantiomeric pure or not [68].

### 4.3. Safety of CPE

Until now, only a few compounds have been approved as permeation enhancers for clinical use since the majority present skin irritation and/or toxicity. The balance between potency and safety is probably the biggest challenge, and no close relationship between these two factors seems to exist [64,71,72,73,74]. Certain mechanisms of action tend to be safer than others. Compounds that usually extract lipids from the bilayer are associated with keratin denaturation, leading to skin irritation. Non-ionic compounds are usually less toxic than ionic ones. Furthermore, when dealing with non-natural amphiphiles, labile bonds confer enhanced biodegradability and thus lower toxicity. Since most of the metabolic activity in the skin only occurs in the viable epidermis, the enhancer can exert its effect in the SC before being hydrolyzed into non-toxic compounds as it reaches the lower layer. Besides biodegradable enhancers, another strategy to improve potency and toxicity profiles is the use of mixtures of enhancers, where the concentrations of each compound can be reduced, and consequently, the toxicity is minimized [64,75]. Moreover, such mixtures may exhibit synergistic effects to improve drug transport across the SC. Common mixtures that present synergistic effects are solvent mixtures (water, alcohols, and fatty acids), eutectic mixtures, vesicles (liposomes, niosomes, and ethosomes), microemulsions, and inclusion complexes (cyclodextrins).

## 5. Amino Acid-Based Enhancers

Amino acids are biomolecules that can fulfill different roles in TDD. Free amino acids display low permeation coefficients and can therefore not be administered transdermally in sufficient amounts to cause any therapeutic effect. However, when assembled into peptides with penetration capacities or transformed into amphiphilic derivatives, they represent one of the most promising groups of permeation enhancers.

Amino acid-based enhancers, except for glycine, have at least one chiral center. They are usually small and contain several polar groups capable of hydrogen bonding. Free amino acids and peptides are known humectants and moisturizers responsible for the natural moisturizing factor (NMF) of the SC [76]. Thus, the use of amino acid-based CPE can indirectly bring a higher amount of water into the SC, increasing permeation even further.

**Figure 4:**
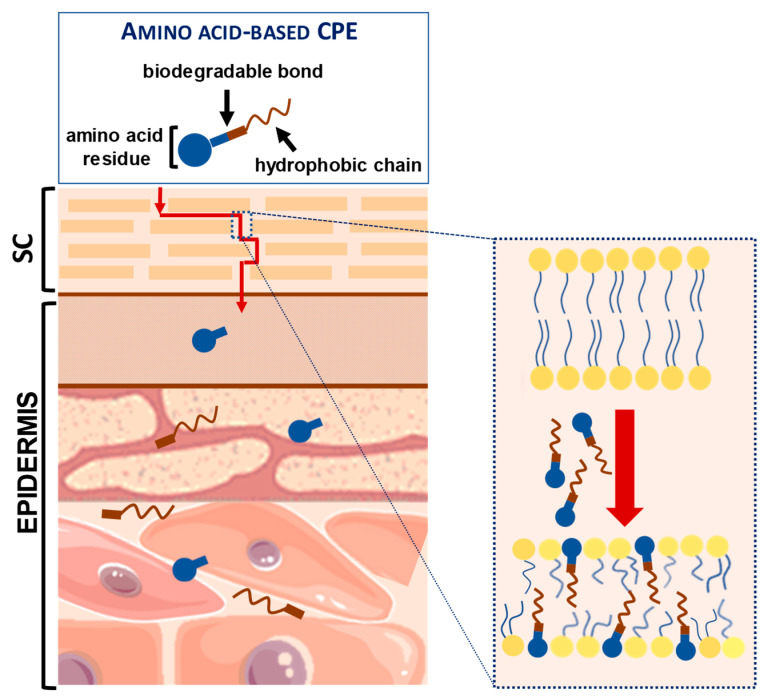
Amino Acid-Based Transdermal Penetration Enhancers.

Amino acid-based amphiphiles possess an amino acid residue as a polar head group linked to a hydrophobic alkyl chain, most frequently through an ester or amide bond. These amphiphiles tend to incorporate into the lipid domain of the SC and disturb its ordered arrangement. When reaching the viable epidermis, the labile ester/amide bond of the amphiphiles may be enzymatically hydrolyzed, giving rise to non-toxic products, with a low irritation potential (Figure 4) [30,77].

Several peptides have also been identified and evaluated as permeation enhancers. These include cell-penetrating peptides (CPPs) [10,78,79] and antimicrobial peptides (AMPs) [80]. CPPs are amphiphilic peptides of up to 30 amino acids derived from natural or unnatural protein sequences, mostly composed of positively charged amino acids, like arginine and lysine [78]. CPPs are commonly linked to the drug (cargo) through covalent or electrostatic bonds and can penetrate the SC corneocyte cells by destabilizing the intercellular matrix and increasing permeability and therefore enabling the transport of their cargoes across the skin. Compared to classic amino acid-based CPEs, peptide-based transport offers some advantages, namely the ability to transport large hydrophilic molecules like proteins, peptides, even nucleic acids, and siRNA, across the skin [10,78]. CPPs can also interact with drugs in different ways, such as through non-covalent interactions in donor formulation, by conjugation with drug components through biodegradable covalent bonds, or through the formation of nanocarriers like liposomes or nanoparticles to be used as drug vehicles. However, most CPPs are covalently linked to their cargo through disulfide linkage, for example, which can significantly alter the biological activity of the transported molecules [78]. Antimicrobial peptides (AMP) contain less than 60 amino acids, cationic and hydrophobic in nature. Thus, they are positively charged amphiphilic molecules and may interact with the anionic lipid bilayers of the SC, thereby enhancing the permeability of drugs.

The use of peptides (CPP and AMP) as chemical permeation enhancers will not be addressed in this review.

In Table 2 are summarized amino acid-based enhancers representative of the different classes of these compounds described in the literature. The chemical structure, the permeation enhancement data, the type of drug permeated, and the toxicity of the compound are also listed for comparison purposes. These kinds of enhancers have been used to promote a higher absorption through the skin of different classes of drugs, including anti-inflammatory steroids (hydrocortisone), nonsteroidal anti-inflammatory drugs (indomethacin), anti-virals (adefovir, tenofovir), antineoplastics (fluoroazuracil), antibiotics (metronidazole), and local anesthetics (tetracaine, ropivacaine). It should be mentioned that tetracaine, hydrocortisone, and indomethacin are considered hydrophobic drugs, while theophylline, adefovir, tenofovir, fluorouracil, metronidazole, and ropivacaine are considered hydrophilic.

In many of the studies performed, Azone^®^ was used as a positive control. Azone^®^ was the first molecule specifically designed as a skin permeation enhancer. It is highly lipophilic (log P ≈ 6.2), shows a strong enhancement capacity and low toxicity [84].

### 5.1. Free Amino Acids

In earlier times, amino acids were studied for their transdermal behavior, spurred by the demand of the pharmaceutical and cosmetic industries to administer peptides, proteins, and amino acids as moisturizing agents in their formulations. Ruland and Kreuter (1991) performed a study on the transdermal behavior of the 20 amino acids in order to assess their in vitro skin permeation ability as well as to estimate an eventual skin reservoir of amino acids [85]. They designed the experiments to distinguish between the charged and uncharged forms and therefore determined the permeation rates of the amino acids at the respective isoelectric point and at physiological p*H*. The permeation coefficients obtained were much lower than those observed for alcohols and were in the range of 10^−5^ cm h^−1^. The somewhat higher permeation rates observed for arginine and lysine at the respective p*I* were attributed to the possible destruction of the skin membrane at these higher p*H* values. Accumulation of the hydrophilic amino acids within the skin was also not observed, as they do not have a high solubility in the lipophilic SC. The authors could not find any relationship between either molecular weight or lipophilicity and permeation rates. The low permeabilities displayed do not advocate the transdermal administration of amino acids *per se*. Sznitowska et al. (1993) also evaluated aspartic acid, histidine, and lysine for their permeation abilities [86]. The results were in agreement with those obtained by Ruland and Kreuter. The experiments were performed at different p*H*, but the ionization state of the amino acids did not significantly alter their permeabilities. Based on the data obtained, the authors suggested a tortuous porous mechanism of transport.

These studies showed that amino acids *per se* were not able to permeate the skin in sufficient amounts to be useful for transdermal administration.

### 5.2. Non-Ionic Amphiphiles

According to the LPP theory formulated by Barry [28,32], an efficient enhancer should be amphiphilic to interact with the lipophilic as well as the hydrophilic components of the SC. Much effort has been made to synthesize such molecules using natural structural motifs and analogs thereof as synthons in an attempt to target the safety of the compounds. Amino acid-based amphiphiles, especially those with biodegradable ester bonds, represent a promising class of enhancers with low toxicity, low skin irritation, and high and reversible activity.

The first report of amino acid derivatives used as absorption enhancers dates back to 1984 and refers to a European patent, by Fix and Pogany [87], on lysine esters of fatty alcohols as absorption enhancing agents for rectal and gastrointestinal absorption.

In 1989, Wong and co-workers [56] designed biodegradable compounds based on amino acid esters to minimize cytotoxicity and irritation potential in an attempt to mitigate the side effects of the first established skin permeation enhancer Azone^®^, namely the skin irritation after long exposure. Several alkyl *N*,*N*-dimethylamino acetates (glycine derivatives) were evaluated for their penetration enhancement, toxicity, and irritation potential. Compounds with 8, 10, and 12-carbon long alkyl chains were shown to enhance permeation of indomethacin (across shed snake skin) by a factor of 2.5, 3.8, and 2.0, respectively, in comparison to Azone^®^. Furthermore, for dodecyl *N*,*N*-dimethylamino acetate (DDAA), no skin irritation and low toxicity were observed. The mechanisms underlying the drug permeation activity of DDAA involve the SC proteins and lipids. In the same study, the authors verified that a bulkier polar headgroup had a negative effect on enhancement potency. The efficiency of DDAA for the permeation of hydrophilic and lipophilic model drugs across human skin was confirmed by Hirvonen et al. (1991) [57]. The promising results obtained led to the development of new biodegradable compounds designed to further improve enhancement rates. In 1993, Doležal et al. performed the synthesis of a series of ε-aminocaproic acid esters and evaluated their potential as permeation enhancers [58]. These derivatives may be viewed as acyclic Azone^®^ analogs with the rigid ring structure replaced by a flexible chain (5 methylene groups between the amine and ester functionalities) and the amide bond by an ester bond to guarantee biodegradability. The authors focused their study on two of the derivatives, octyl-6-aminohexanoate (OCEAC, OCtyl-Ɛ-AminoCaproic acid ester) and dodecyl-6-aminohexanoate (DDEAC, DoDecyl-Ɛ-AminoCaproic acid ester). Both compounds exhibited excellent permeation enhancement for theophylline in the three vehicles tested (oil, water, water-propylene glycol), with flux rates across the skin about seven times higher than for Azone^®^. The compounds exhibited slightly lower toxicity than Azone^®^ and no acute dermal irritation. Further studies revealed that this enhancer is only active after the capture of CO_2,_ yielding the active structure transkarbam 12 [88].

In 1993, the synthesis and enhancement potential of another amino acid derivative, dodecyl-2-(dimethylamino)propionate (DDAIP), the first amino acid-based enhancer (alanine derivative) to be patented and commercialized, was also reported [89]. Compared to Azone^®^, DDAIP was 4.7 times more active in the permeation enhancement of indomethacin. Direct comparison of DDAIP with DDAA using 5-fluorouracil as a model drug revealed an almost 3-fold enhancement in favor of DDAIP, which was remarkable, given the only difference between the two compounds is a methyl group in the amino acid residue. In 2004, the mechanism of action of DDAIP was investigated by Wolka et al. using differential scanning calorimetry (DSC) [90]. The results indicated that permeation enhancement promoted by DDAIP involves interaction with the polar region of the phospholipid bilayer as well as with the hydrophobic region, increasing the fluidity of lipid hydrocarbon chains. DDAIP is well tolerated by the skin and exhibits low toxicity, as the presence of the labile ester bond in DDAIP enables its easy breakdown (by esterases) into non-toxic dimethylalanine and dodecanol.

More recently (2009), Novotný and co-workers [61] studied a series of *N*,*N*-dimethylamino acid esters, analogs of DDAIP, as transdermal permeation enhancers, with the purpose to evaluate the effects of chirality, chain length between amino and ester groups, and polyfluorination, on their enhancement potential. Four model drugs with distinct physicochemical properties were used. From this study, the authors concluded that chirality has no influence on the activity of the enhancer. On the other side, the substitution of the hydrocarbon chain by a fluorocarbon chain led to a complete loss of activity. Concerning the chain between the amino and ester groups, linear chains with four to six carbon atoms conferred the best penetration enhancing capacity, although, for shorter and longer chains, only a slight reduction in activity was observed. On the other hand, the substitution of the “branched” chain in DDAIP by a linear ethylene linking chain to yield its β-alanine isomer led to an almost 3-fold increase in theophylline flux. A detailed comparison of one of the compounds under study, namely dodecyl 6-(dimethylamino)hexanoate (DDAK, Table 2), with the parent compound DDAIP was performed. DDAK was shown to be a more potent enhancer for three of the drugs (theophylline, hydrocortisone, and adefovir) tested, while DDAIP was superior for indomethacin. The higher potency of DDAK was attributed to its higher lipophilicity and flexibility, which may enable DDAK to adopt an optimal conformation and thus better interact with the SC lipid bilayers. DDAK was rapidly metabolized by esterases, presented low toxicity and reversible action, and is considered until today one of the most promising amino acid-based enhancers for future clinical and cosmetic applications.

Apart from these glycine and alanine analogs, several other amino acid amphiphiles were synthesized and evaluated for permeation enhancement, using different amino acid moieties, such as, proline [91,92,93,94], serine [49,60,91,93], lysine [87,95,96], sarcosine [58,91], among others.

Fincher et al. (1996) evaluated a series of *N*-dodecanoyl-amino acid methyl esters (glycine, serine, proline, valine, alanine, tryptophan, leucine, phenylalanine, tyrosine, and methionine derivatives) as permeation enhancers using hydrocortisone (HC) as the model drug [93]. All of the compounds increased the permeation of HC but to a lesser extent than Azone^®^. The best performing compound was the proline derivative, which happens to be the structurally most similar compound to Azone^®^, but serine and alanine derivatives also performed well. Proline derivatives were also studied by Tenjarla and co-workers (1999) [94]. These authors synthesized several *N*-acetylprolinates with varying chain lengths (C5-C18) and evaluated their permeation enhancing capacities using two model drugs, benazepril, and hydrocortisone. For both drugs, the best enhancers were the C11, C12, and oleyl derivatives. All the compounds, except C5 and C8, enhanced the permeation of benazepril, although to a lesser extent than Azone^®^. In the case of HC, an increase in drug flux was observed with all the enhancers. The C11, C12, and oleyl derivatives were indeed more active than Azone^®^. The better results obtained with C11 and C12 derivatives are in agreement with those obtained for other classes of enhancers (e.g., fatty acids and alcohols) [97]. The high enhancement rate of the oleyl derivative has already been explained to result from the kink it impairs to the alkyl chain, which may affect the packing of the lipid bilayer of the SC. The authors performed DSC studies to infer the mechanism of action of the enhancers. They concluded that these compounds most probably interact with the lipid domain of the SC, disturbing the lipid packing and increasing lipid fluidity. From the results obtained by Fincher and Tenjarla on the proline derivatives, the position of the lipophilic alkyl chain seems to have an important effect on the permeation profile of the resulting CPE. In fact, relative to HC, the two isomers present different permeation enhancement when compared to the positive control, Azone.

Vávrova et al. [60] in 2003 conducted a study involving double-chained glycine and serine derivatives to mimic ceramides, the most prevalent lipids in the CLE. The authors hypothesized that the similarity of the enhancers to the ceramides present in the SC might have a positive influence on their permeation enhancing properties. The best performing compound was the alanine derivative with two 12-carbon saturated alkyl chains, which was five times more active than Azone^®^ (12.5-fold enhancement relative to control) for the permeation of theophylline. The presence of the hydroxymethyl group in the serine derivative significantly reduced the permeation enhancing capacity (2.7-fold relative to control), indicating a negative effect of the hydrogen bonding ability. The introduction of a *cis*-double bond in one of the alkyl chains also decreased the activity. This behavior was somehow unexpected, as the conformational kink in the oleyl chain was reported to enhance permeation activity due to fluidization of the lipid domain [70,94]. The use of two amino acids in the headgroup (dipeptides) led to a lower activity as well. This was explained by the increased difficulty of the compounds to incorporate between the hydrophobic chains of the SC ceramides due to the bigger volume of their polar groups. To further investigate the influence of the different parameters on the enhancement ability of this type of enhancers (ceramide analogs), a QSAR study involving a larger set of compounds was performed [60]. The results obtained confirmed the negative effect of the hydrogen bonding ability on the enhancing capacity of this type of compound. The polar head of the enhancers seems to be responsible for their permeation into the SC and translocation to the site of action, but it does not influence the mechanism of action. Disturbance of the lipid packing of the intercellular lipid domain is attributed to the hydrophobic alkyl chains, whose lengths are the most important parameter. Yet, the same authors reported the barrier repairing capacity of another serine-derived ceramide analog, 14S24, possessing 14-carbon and 24-carbon saturated alkyl chains [98]. In 2013, in another study conducted in the same laboratory (Janůšová et al. [91]), the permeation enhancing capacities of several biodegradable single- and double-chained amino acid amphiphiles, based on proline, sarcosine, alanine, and glycine, were compared, using TH and HC as model drugs. The double-chained derivatives, designed to resemble ceramides, exhibited no enhancing effect, whereas the single-chained ones significantly increased skin permeation, being in some cases superior to positive controls Azone^®^, DDAK, and DDAIP. The results again showed the best performance for 12C-alkyl chains and also confirmed the negative effect of hydrogen donner bonding on enhancing activity. In fact, the best performing enhancers were the single-chained proline and sarcosine derivatives, which are both disubstituted amides, and thus only hydrogen bond acceptors [60]. A derivative of proline with a tertiary *N*-methyl group instead of acetyl was also synthesized, to mimic DDAK and DDAIP. This change resulted in a decrease in activity, which was expected due to the reduction in the number of H-bond acceptor sites. The stereochemistry was shown to have no influence on the activity of the enhancers since for proline and alanine, no differences between L- and D-enantiomers were observed. From this study, the best enhancer was the L-proline derivative with an ester bond to an alkyl chain of 12 carbon atoms and a *N*-acetyl group (L-Pro2, Table 2). L-Pro2 increased the solubility of HC in the donor sample, which may, in part, account for its higher enhancing activity. This enhancer presented reversible action, positive synergy with propylene glycol, IC_50_ values to human dermal cell lines lower than those of some commercially used enhancers, and proved activity and safety in in vivo models, specifically in rats. These results were consistent with previous studies, which demonstrated proline-based amphiphiles to be potent CPEs [94].

The use of different alkyl chain lengths in amino acid-based amphiphilic derivatives has been vastly reported in the literature [48,67,68]. However, reports on amphiphilic derivatives with unsaturated chains are rather scarce. In 2018, Rambharose et al. reported the synthesis of mono-, di-, and tri-fatty acid esters of β-alanine, to be used as CPEs [81]. The influence of the degree of unsaturation of the alkyl chain as well as of the number of alkyl chains present at the head group level was evaluated. Therefore, different fatty acids with different unsaturation degrees (oleic acid, linoleic acid, and linolenic acid) were used. Unsaturated fatty acids are a well-known category of CPE, that are able to disrupt the packed structure and form separate phases within the intercellular lipids, thus decreasing either the resistance or the length of the diffusional path [99]. The results obtained by Rambharose and co-workers showed that β-alanine “fatty” esters have, in fact, higher enhancement capacity than the corresponding free fatty acids. All the compounds studied increased the drug flux of tenofovir across the skin. The monooleate ester derivative (Table 2) at a concentration of 1% displayed the best permeation enhancing activity for the anti-viral drug tenofovir, with an enhancement rate of six compared to tenofovir alone, without any toxic effects.

In 2019, Kopečná et al. [100] developed terpene-amino acid enhancers using 6-(dimethylamino)hexanoic acid as a polar headgroup. Terpenes, hydrocarbon-based compounds composed of one or more isoprene units, are a well-known class of CPE with strong and generally safe enhancement capacities [101,102,103,104]. A great diversity of compounds can be obtained by conjugation of amino acids with terpenes due to the chemical diversity of these hydrocarbons: cyclic or acyclic structures, different levels of unsaturation, and presence of one or more hydroxyl groups. In this study, the authors designed new permeation enhancers based on the previously reported amino acid derivative DDAK. They substituted the dodecyl chain in DDAK with different terpene alcohols, such as citronellol, nerol, borneol, farnesol, among others. The compounds were evaluated for their ability to permeate theophylline, TH, and hydrocortisone, HC, through human skin. Among all derivatives, the citronellol derivative citronellyl 6-(dimethylamino)hexanoate (C-DAK) showed exceptional results, increasing 47 times the TH flux and 56 times the HC flux, relative to control (no enhancer). Its performance was very similar to that of DDAK (42 for TH and 57 for HC). The cinnamyl alcohol ester Ci-DAK displayed the strongest permeation enhancing activity for HC, with an ER of 82, which was 1.5 fold the effect of DDAK, but had no impact on TH permeation. Borneol derivative B-DAK also displayed enhanced permeation activity for both drugs, with ER values of 11 and 45 for TH and HC, respectively. C-DAK, Ci-DAK, and B-DAK presented very low cytotoxicity to human dermal cell lines, similar to DDAK. The terpene esters had, in most cases, higher efficacies than the corresponding terpene alcohols. Their effects on the skin barrier properties were reversible and most probably involved intercalation into the hydrophobic region of the lipids of the SC, perturbing their arrangement and leading to their fluidization. The C-DAK derivative showed the most interesting profile, being a potent CPE that may be used for the transport of drugs that otherwise struggle at crossing biological barriers.

### 5.3. Surfactants

Amino acid-based surfactants possessing cationic and anionic charge have also been used in transdermal delivery approaches. Ionic surfactants have strong lipid solubilizing abilities and protein denaturing capacities and have been widely studied as chemical permeation enhancers [105,106]. However, these charged molecules are often associated with cytotoxic effects on skin cells. Gemini surfactants are known to have enhanced interfacial properties when compared to their monomeric analogs. They present lower critical aggregation concentration (cac) values than monomeric analogs and a more versatile phase behavior [107]. Gemini structures were shown to possess better cytotoxic profiles, and some of them were claimed to be promising candidates for permeation enhancement [48,108]. When derived from natural molecules, such as amino acids, ionic surfactants present reduced cytotoxicity and high biocompatibility [109]. Glutamic acid-based based amphiphiles, for example, are anionic surfactants with acceptable biocompatibility [110]; serine-based cationic surfactants also display low toxicity [111,112]. Due to their physicochemical properties and self-assembling behavior, amino acid-based gemini surfactants can be used as skin permeation enhancers as well as drug delivery vehicles in TDD [49,95,96]. Hikima et al. (2013) used a peptide gemini amphiphilic molecule (sodium dilauramidoglutamide lysine, DLGL) to evaluated it as a CPE for the delivery of L-ascorbic acid 2-glucoside (AAG), a hydrophilic compound of interest for cosmetic applications [113]. DLGL is a tripeptide (glutamic acid-lysine-glutamic acid) with two dodecyl chains and can be classified as a gemini surfactant. Sodium lauramidoglutamide, LG, was also evaluated for comparison purposes as the corresponding monomeric counterpart. DLGL and LG enhanced penetration flux of AAG by a factor of 13 and 69, respectively. However, DLGL allowed higher skin accumulation of AAG than LG (22% vs. 8%). The presence of two alkyl chains is known to decrease the flux of gemini surfactants, as their mobility in the lipid bilayer is reduced. The results suggest that DLGL may be immobilized in the SC and preserve the hydrophilic drug in the skin. On the other side, LG, as it can easily move through the lipid bilayer of the SC, might create pathways through the skin and penetrate across it with AAG.

Teixeira et al. (2014) tested lysine-based double-chained anionic and non-ionic surfactants with varying alkyl chain lengths as CPEs for tetracaine and ropivacaine hydrochloride (local anesthetics) [95]. The highest enhancement rate for tetracaine permeation was obtained with the 16-carbon non-ionic and anionic double-chained derivatives (ER = 1.33 and 1.27, respectively). In the case of ropivacaine hydrochloride, all the anionic surfactants increased permeation, whereas the non-ionic surfactants had no effect on permeation. In this case, the most active enhancer was the 16-carbon anionic double-chained derivative, which displayed a 6-fold increase in the flux of the hydrophilic drug. The high enhancing effect was attributed to favorable electrostatic interactions between the anionic surfactant and the cationic drug. Both anionic and non-ionic surfactants displayed no cytotoxicity to HEK cell lines at the tested conditions. The same authors (2015) also evaluated serine-based gemini surfactants, with varying hydrocarbon chain lengths and headgroup charges, as CPEs for the same drugs [49]. They performed in vitro permeation studies and molecular dynamics simulations to rationalize the results obtained. A DPPC bilayer was used as a simple model of the skin lipids organization. The most effective enhancer for both drugs was found to be the cationic surfactant with the longer alkyl chains (14C) and a five carbon spacer (ER = 3 and 1.9 for ropivacaine and tetracaine, respectively, Table 2). Compounds with ten carbon spacers showed, in general, lower or even no permeation enhancement. As expected, the non-ionic surfactants were less toxic to HEK cells than the cationic ones, although for concentrations up to 16 μM no negative effect on cell viability was observed for any of the CPE. Thus, these serine-based surfactants show promise as candidates for transdermal drug delivery, especially for hydrophilic drugs, which are generally poorly absorbed through intact skin.

Muzzalupo, R. et al. (2017) reported the use of a cationic gemini lysine-based surfactant as a drug delivery system for the topical administration of tetracycline [96]. The gemini surfactant displayed low cytotoxicity, low hemolytic activity, and high biodegradability. The surfactant self-assembled into vesicles, which were loaded with the drug, to assess its potential as a carrier that confers permeation improvement across membranes. The loaded vesicles can enhance TDD through different mechanisms: They can disaggregate and release the drug before coming in contact with the SC, and the resulting surfactant monomers act as chemical permeation enhancers. The vesicles can also be absorbed by the SC and mix the surfactant molecules within the lipid bilayers of the SC, releasing the drug. This behavior causes skin permeation enhancement through lipid fluidization, but it also improves drug partitioning. Finally, the vesicles can deform and go intact through the SC, reaching the systemic circulation and releasing the drug in the blood. The authors proved that the cationic vesicles behave as drug permeation enhancers after topical application but also allow sustained and controlled drug delivery in the case of parenteral administration. Amino acid-based vesicles have a great potential for TDD and can be used for the encapsulation of hydrophilic as well as lipophilic drugs. A hydrophilic drug will be encapsulated in the core of the vesicle, whereas a lipophilic drug will be entrapped between the lipid bilayers, made of the nonpolar tails of the surfactant molecules.

### 5.4. Gelling Agents

Gel forming agents generally produce a semisolid consistency in a formulation, thus increasing the resident time at the site of administration. Examples of gelling agents include acrylic acid-based polymers, gums, and cellulose derivatives.

Dahlizar et al. (2018) described the design of a gel spray formulation using *N*-palmitoyl-glycine-histidine (Pal-GH, Table 2) and incorporated ivermectin (IVM) a hydrophobic drug used to treat scabies [114]. IVM concentrations in viable epidermis and dermis (VED) were increased after topical application of the formulation containing propylene glycol. However, no skin permeation was observed. This approach may be used to increase skin concentration of otherwise non-absorbed drugs from topical formulations. The same authors described the use of a gelator made up of the same alkylpeptide Pal-GH, to increase the skin permeation of metronidazole (MTZ), a hydrophilic drug with low skin permeability [82]. Formulations of Pal-GH with MTZ alone and in the presence of several CPEs were prepared. Aqueous solutions of MTZ with and without CPEs were also prepared for comparison. The skin permeation of MTZ was enhanced in the Pal-GH formulation. This increase was rationalized in terms of fibrous micelle-like Pal-GH assemblies formed, which may fuse with the lipid bilayer, fluidizing the SC and increasing diffusion of the drug. The presence of some of the CPEs studied (isopropyl myristate and propylene glycol) in the gel formulation had a synergistic effect on MTZ skin permeation, most probably through an increase in the drug partition from the gel into SC. This amino acid-based formulation may thus enhance the skin permeation and concentration of several drugs and has a promising potential for use in dermatological formulations.

### 5.5. Ionic Liquids

Ionic liquids (ILs) are molten salts composed of a large organic cation and an organic or inorganic anion. They possess high thermal and chemical stability, high solubilizing capacity and are widely used in pharmaceutical applications. ILs were shown to enhance transdermal permeation through mechanisms involving disruption of cell integrity, lipid fluidization and extraction, and several reports on their use in topical emulsions to promote drug solubility and permeation are available [115,116,117]. Recently, Moshikur, R and co-workers (2020) developed a new family of IL-based on fatty acids and amino acid esters [118]. Fatty acids and amino acid esters are well-known potent CPEs with low cytotoxicities. In this work, oleic and linoleic acid were combined with different amino acid ethyl esters (β-alanine, leucine, and proline) to create different IL. Their physicochemical properties were determined, and they were then used as solvents in the drug donor phase (to increase solubility) and as CPE at the same time. An increase in the solubility of ibuprofen in the IL was observed and attributed to multiple hydrogen-bonding interactions between drug and IL. The L-proline ethyl ester linoleate IL exhibited the best results among all derivatives and was capable to significantly increase the permeation of ibuprofen through a mechanism involving lipid fluidization of the SC. The same IL also displayed a high permeation enhancement for fluorescein-isothiocyanate (FITC). The capacity of delivering macromolecules through the skin is an important achievement for TDD because it has been one of the most difficult obstacles to overcome. Jesus et al. (2019) also synthesized amino acid-based ILs, combining anionic *N*-acetyl amino acid derivatives with cationic *N*-alkyl choline derivatives [119]. The goal was to use these biocompatible ILs as co-solvents to enhance the solubility of highly hydrophobic drugs in aqueous environments. The new ILs showed significant solubility improvements for paracetamol and sodium diclofenac with low cytotoxic effects on human dermal cell lines. These results proved that the application of these ILs as co-solvents in donor formulations for both oral and dermal delivery has positive effects on drug availability. In another study, Zheng et al. (2020) synthesized and evaluated IL based on glycine, L-proline, and L-leucine (Table 2) esters with different carbon chain lengths (C8 and C12), for the permeation of 5-fluorouracil (5-Fu) and hydrocortisone (HC) [83]. The proline and leucine dodecyl ester hydrochlorides displayed the best activities. These amino acid-based IL interact with the intercellular lipid domain by lipid fluidization and lipid extraction, improving the permeation of both hydrophilic and hydrophobic drugs greatly.

Overall, amino acid-based ionic liquids show good biocompatibility and are potential candidates as novel CPE for TDD.

## 6. Conclusions

This review summarizes the up-to-date available research on skin permeation enhancers based on amino acids for application in medicine. The discussion provided in this review is intended to be clear but not exhaustive, representing the general strategies involving amino acid-based chemical enhancers, their mechanism of action as far as known, and the main results obtained in terms of toxicity and efficacy of the compounds as CPEs.

The recent development of amino acid-based enhancers shows bright possibilities for the future of TDD methods. Amino acid-based enhancers combine versatility, potency, and safety features unmatched by commonly used CPEs. Their structural versatility enables the combination of multiple mechanisms of action, such as skin permeability enhancement or drug solubility improvement. However, the mechanisms of action of CPEs are not yet fully understood, but the development of new analytical methods over the last decades has shed some insight into this field, and their total elucidation is just a question of time. The knowledge of the way an enhancer interacts with the SC is of the utmost importance for the design of novel efficient drug delivery formulations.

The potential of amino acid-based enhancers may be further explored through the combination with other enhancers, be it other chemicals or physical methods, where a synergistic enhancement effect can be expected. Such an approach may lead to the long-awaited success of this drug delivery method. In fact, in order for TDD to truly surpass other drug delivery methods, it needs to be available for the delivery of a broader variety of molecules, especially hydrophilic drugs and drugs with high molecular mass, that constitute the majority of new drugs and vaccines recently discovered and approved.

## Figures and Tables

**Table 1:** Examples of CPEs.

Solvents	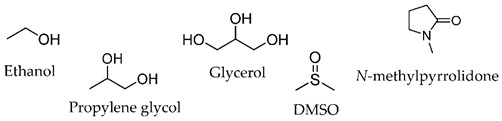
Fatty acids and alcohols	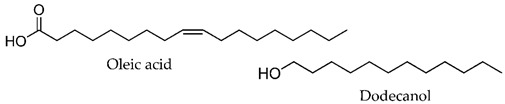
Lactams	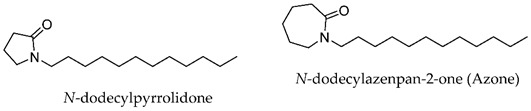
Terpenes	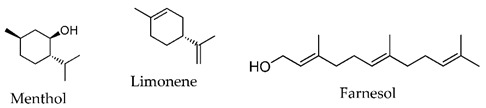
Sugar and vitamin derivatives	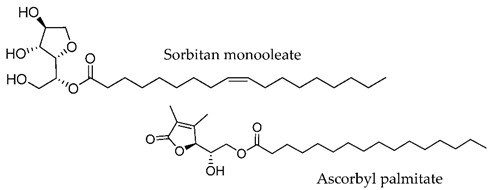
Esters	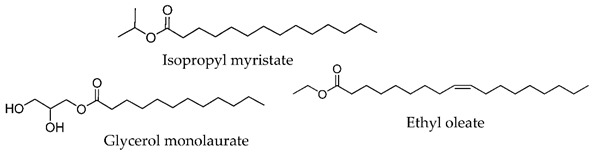
Amino acid derivatives	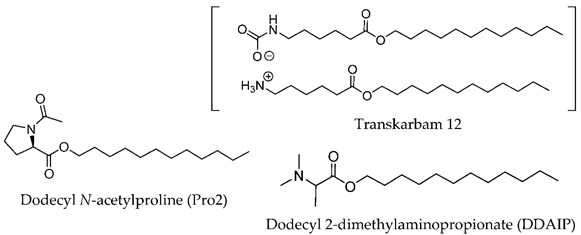

**Table 2:** Examples of amino acid-based enhancers.

Polar Head Amino Acid	Enhancer Structure	ER	Flux Rate /µg·cm^−2^·h^−1^	Drug	Donor Conditions	Cytotoxicity		Ref
6-(Dimethylamino)hexanoic acid	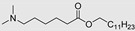 DDAK	17.8	42.2 ± 14.3	Theophylline	5% drug in 60% PG	75.6 ± 12.7 µM IC50—HaCaT	175.2 ± 27.6 µM IC50—3T3	[61]
		43.2	4.78	Hydrocortisone	2% drug in 60% PG			
		13.6	19.0	Adefovir	2% drug in PB pH 4.8			
		-	8.7	Indomethacin	2% drug in 60% PG			
**L-Proline**	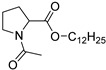 **L-Pro2**	40.0	70.3 ± 7.7	Theophylline	5% drug in 60% PG	68.2 ± 11.5 µM IC50—HaCaT)	182.6 ± 6.7 µM IC50—3T3	[60]
		47.0	6.54 ± 0.87	Hydrocortisone	2% drug in 60% PG			
**β-Alanine**	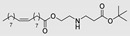	5.87	240.55 ± 21.06	Tenofovir	2% drug in 4 % HPMC	80 % viability for Hep G2, MCF 7 and A549 cells, at 100 µg/mL		[81]
**Serine**	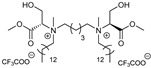	1.87	37.08 ± 1.85	Tetracaine	2.5% drug in 1 % HPMC and HP-β-CD	100 % HEK cells viability at 0.14 µM		[49]
		2.96	5.74 ± 0.74	Ropivacaine	2.5% drug in 1 % HPMC			
**Glycine-Histidine**	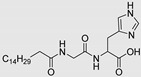 **Pal-GH**	7.2	17.7 ± 1.3	Metronidazole	1% drug in gel formed with 5 % enhancer	-------------------------		[82]
**L-Leucine**	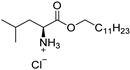	7.14	851.47 ± 69.49	Fluorouracil	0.5% drug in water	177 ± 14 µM TC50—HaCaT	387 ± 19 µM TC50—3T3	[83]
		2.28	29.92 ± 5.33	Hydrocortisone	0.1% drug in water			
